# Monolayer culture of intestinal epithelium sustains Lgr5^+^ intestinal stem cells

**DOI:** 10.1038/s41421-018-0036-z

**Published:** 2018-06-12

**Authors:** Yuan Liu, Zhen Qi, Xintong Li, Yanan Du, Ye-Guang Chen

**Affiliations:** 1 The State Key Laboratory of Membrane Biology, Tsinghua-Peking Center for Life Sciences, School of Life Sciences, Tsinghua University, 100084 Beijing, China; 2 Department of Biomedical Engineering, School of Medicine, Tsinghua University, 100084 Beijing, China

Dear Editor,

The self-renewing intestinal epithelium is functionally organized into proliferating crypts and differentiating villus^1^. At the bottom of the crypts, Lgr5^+^ intestinal stem cells (ISCs) reside between terminally differentiated Paneth cells and actively drive the self-renewal of intestinal epithelium^1, 2^. The balance between proliferation and differentiation of Lgr5^+^ ISCs is critical for the intestinal homeostatic maintenance^1^. The establishment of the three-dimensional (3D) self-renewing organoid culture system in Matrigel supplemented with the growth factors EGF, Noggin and R-spondin (ENR) provides an ideal system to dissect how stem cells integrate multiple signals to maintain stemness and undergo malignant transformation^3^. However, the 3D geometry nature of organoids prevents visual tracing of stem cells and challenges the dissection of interaction between stem cells and niche cells with microscopy. Much effort has been made to establish two-dimensional (2D) culture of intestinal epithelial cells, but whether these systems are suitable for adult Lgr5^+^ stem cell study remains elusive^4–6^. Thus, it would be of great value to establish *a bona fide* 2D monolayer culture system to study Lgr5^+^ ISCs and facilitate drug screening. Also, it is interesting to determine whether Lgr5^+^ cells are able to maintain stem cell activity on monolayer without the curvature structure.

To establish a 2D monolayer culture system, we first explored whether Lgr5^+^ ISCs could survive and maintain stemness on a matrix-coated plate. Isolated intestinal crypts from *Lgr5-GFP-IRES-CreERT2* mice were re-suspended in the ENR-containing organoid culture medium and seeded on collagen I- or Matrigel-coated plates with or without the non-muscle myosin IIA inhibitor blebbistatin as our previous work showed that blebbistatin could significantly improve the survival of Lgr5^+^ ISCs and the growth of organoids^7^. Although a few cells survived in the absence of blebbistatin, the addition of blebbistatin greatly enhanced the attachment and growth of intestinal epithelial cells on Matrigel-coated plate, much better than Y-27632 (Supplementary Fig. S1A), and supported the survival and growth of Lgr5^+^ ISCs (Fig. 1a). However, no Lgr5^+^ ISCs were observed in the collagen I-coated plates even in the presence of blebbistatin (data not shown). To examine whether uniform thickness would improve the growth of Lgr5^+^ ISCs in 2D culture, we developed a novel method to generate a thin layer of Matrigel with uniform thickness on glass sheets based on our previous work^8^. Briefly, a single layer of Matrigel was formed between a pre-treated coverslip and a hydrophobic glass slide (see Supplementary Methods), and the thickness can be easily controlled by Matrigel volume and coverslip dimension. We found that the 10 μm-thick Matrigel best supported the growth of Lgr5^+^ ISC monolayer, whereas thicker Matrigel layer (50 μm) allowed the formation of an organoid-like 3D structure (Supplementary Fig. S1B). Thinner Matrigel layers such as 5 μm could still support the growth of Lgr5^+^ ISCs, but the efficiency was lower, similar to the Matrigel-coated culture. The bright-field microscopy revealed that the monolayer exhibited heterogeneity in both cell morphology and densities (Fig. 1a). Highly dense compartments with small sized cells were enriched with Lgr5^+^ ISCs, which were surrounded by large differentiated cells. We confirmed the epithelium origin by staining of E-cadherin and ZO-1 and determined the monolayer nature by Z-stack modeling (Fig. 1b, c; Supplementary Fig. S2 and Movie).

**Fig. 1:**
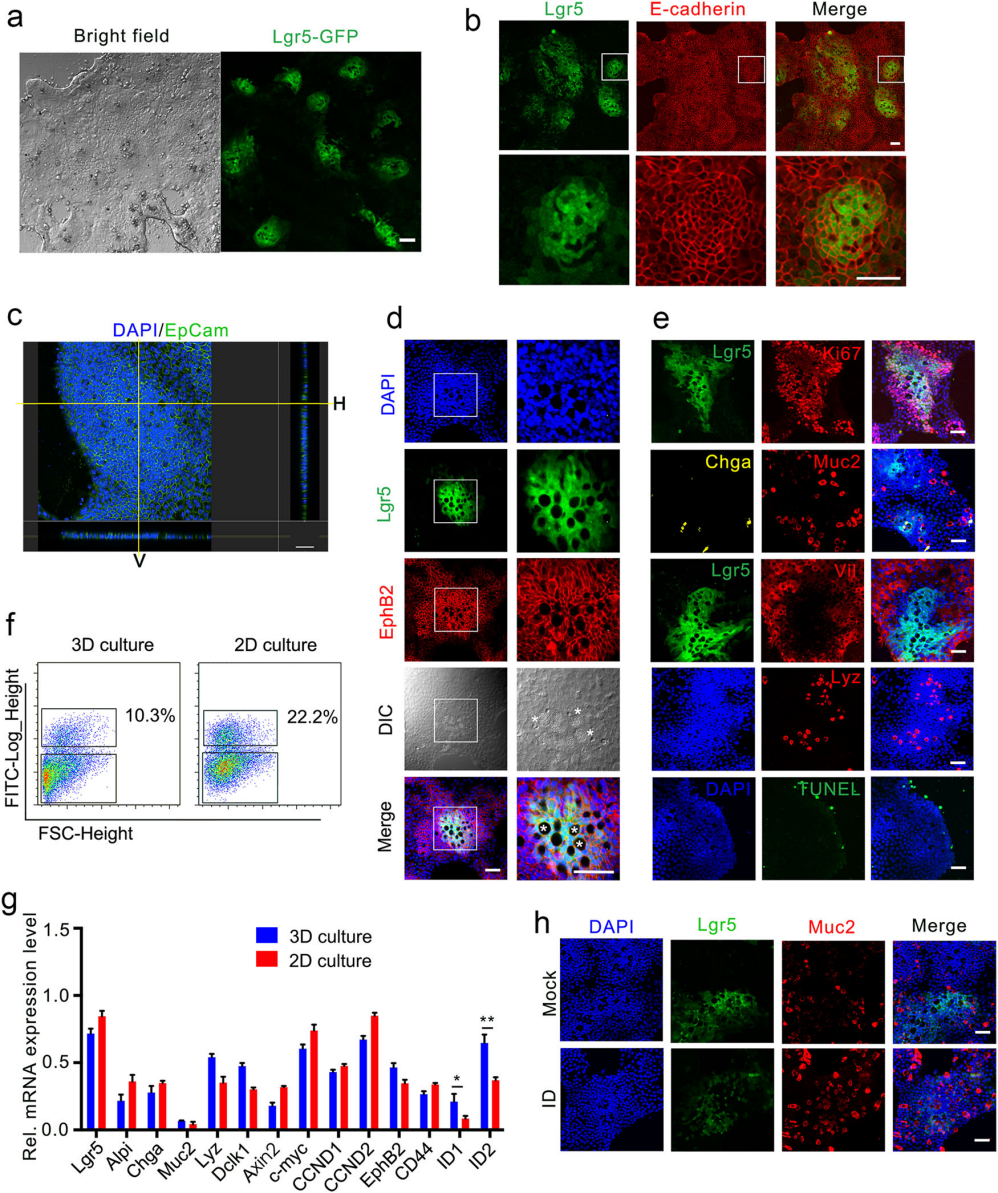
Establishment of a self-renewing 2D monolayer culture of Lgr5^+^ ISCs. **a** Representative bright-field and Lgr5-EGFP fluorescence images of small intestinal epithelial cells cultured in 2D system with blebbistatin, EGF, Noggin, and R-spondin1 (BENR) for 2 days, followed by culturing in BLRC for 4 days. **b** E-cadherin staining of epithelium and GFP of Lgr5^+^ stem cells in 2D-cultured monolayers. **c** Confocal images of monolayers stained for EpCam (green) and DAPI (blue). Right and bottom panels suggest the horizontal (H) and vertical (V) projections. **d** Confocal images staining with EphB2 (red) showed the location of crypt, stem cells and Paneth cells. Asterisks mark the Paneth cells. **e** Confocal images stained for proliferation (Ki67^+^), Enterocytes (Vil^+^), Paneth cells (Lyz^+^), Goblet cells (Muc2^+^), enteroendocrine cells (Chga^+^), and apoptosis (TUNEL^+^). **f**, **g** Representative FACS analysis (**f**) and gene expression (**g**) in 2D or 3D system. The data were analyzed by Student’s *t*-test and shown as mean ± SD. *P < 0.05, **P < 0.01. **h** Muc2 staining of Goblet cells from cells initially cultured in 2D system (7 d) and then transferred to ENR or ENR plus ID (5 d). I: IWP-2. D: DAPT. Scale bars, 50 μm

To better sustain Lgr5^+^ ISCs on the 2D monolayer, we modified the culture medium with small molecules. EGF is dispensable in this system, as removal of EGF only led to a slight decrease of Lgr5^+^ ISCs (Supplementary Fig. S3). Noggin could be replaced by the BMP type I receptor inhibitor LDN-193189 (LDN) after first two days, and the combination of LDN and the GSK-3 inhibitor CHIR-99021 greatly increased the proportion of the Lgr5^+^ ISCs (Supplementary Fig. S3). The BLRC (blebbistatin, LDN, R-spondin, and CHIR) culture medium supported a robust monolayer culture of Lgr5^+^ ISCs without affecting differentiated lineage cells (Fig. 1d–f; Supplementary Fig. S3). With the modified 2D system, we tested the culture efficiency from different portions of small intestine, and found that the survival rate was highest in the duodenum (Supplementary Fig. S4A). Furthermore, the monolayer culture could be applied to APC KO crypts (Supplementary Fig. S4B).

Next, we assessed whether the cells cultured with this 2D system resembled the in vivo intestinal epithelium. We have noticed that in our 2D culture, Lgr5^+^ ISCs were intermixed with visible large Paneth cells, reminiscent of the in vivo crypt base (Fig. 1d). Proliferation marker Ki67 staining indicated that the cells in the stem cell zone marked by Lgr5 were actively proliferating. Other cell lineage markers were expressed in the non-stem cell compartment: villin (enterocytes), mucin 2 (goblet cells), and chromogranin A (enteroendocrine cells) (Fig. 1e, g), indicating that normal differentiation occurs in the monolayer culture. Moreover, the cells at the border underwent apoptosis (Fig. 1e), resembling the in vivo villus tip. Tracing of the progeny of Lgr5^+^ cells from *Lgr5-CreER; loxp-Stop-tdTomato* mice in the 2D culture revealed that Lgr5^+^ ISCs sustained the self-renewal of this monolayer (data not shown).

We next assessed whether this 2D system could resemble the previously established in vitro 3D organoid culture system. The isolated crypts were cultured either in the 3D Matrigel using the ENR-containing medium or on the 2D Matrigel layer with the BLRC-containing medium for 5 days. FACS analysis revealed that the proportion of Lgr5^+^ ISCs in the 2D monolayer culture was significantly higher than the one in the 3D organoid culture (22.2% vs. 10.3%) (Fig. 1f). The Lgr5^+^ ISCs in monolayer exhibited stem cell properties as they efficiently formed 3D organoids in Matrigel (Supplementary Fig. S5A and S5B) and possessed a similar gene expression profile to Lgr5^+^ ISCs from organoids except for ID1 and ID2 (Fig. 1g). To show that the Lgr5^+^ ISCs could be directed to differentiate into specific cell lineage, we changed the BLRC medium to ENR medium, which allows differentiation into mature cell types^9^. Indeed, the combination of the Wnt inhibitor IWP-2 (2 μM) and the Notch inhibitor DAPT (10 μM) efficiently induced goblet cell differentiation (Fig. 1h; Supplementary Fig. S5C, D).

In summary, we successfully established a 2D culture system that could support self-sustaining of Lgr5^+^ intestinal stem cells in monolayer. Importantly, our approach has a number of advantages over the existing in vitro organoid culture system. The 2D culture system provides an intuitionistic method for observation of the dynamics of Lgr5^+^ stem cells and facilitates cell imaging studies. In contrast to the inaccessibility of the apical domain of epithelium cells in organoids, exposure of the apical domain in the monolayer culture also provides a good system for the microbe–epithelial cell interaction study and drug screening.

Note: During the preparation of our manuscript, Thorne and their colleagues reported a similar 2D culture system of intestinal epithelium^10^. Different from their system, we found that the addition of blebbistatin and Matrigel of uniform thickness could better sustain Lgr5^+^ ISCs.

## Electronic supplementary material

Supplementary methods and figures

video legend

3D reconstitution video
