# Supplementary material for: Monolayer culture of intestinal epithelium sustains Lgr5+ intestinal stem cells
Source: Cell Discov. 2018 Jun 12;4:32. doi: 10.1038/s41421-018-0036-z (PMC5997714; doi:10.1038/s41421-018-0036-z)
Supplement: Supplementary file 1 — Supplementary methods and figures [file 41421_2018_36_MOESM1_ESM.docx]

**Supplementary information**

**Monolayer culture of intestinal epithelium sustains Lgr5^+^ intestinal stem cells**

Yuan Liu^1#^, Zhen Qi^1#^, Xintong Li^1^, Yanan Du^2^, Ye-Guang Chen^1^*

**Supplementary Methods**

**Mice.** *Lgr5-EGFP-IRES-creERT2*[1] and *Apc^fl/fl^* mice were obtained from the Jackson Laboratory. *Vil-creERT2* mice[2] were a gift from Sylvie Robine. All mice were back-crossed into the C57BL/6 genetic background for at least 10 generations. The 8- to 16-week-old mice were used for crypt isolation. For gene-ablation experiments, 6- to 8-week-old mice were injected intraperitoneally with 100 μl tamoxifen in sunflower oil at 20 mg/ml for 5 consecutive days. *Apc^-/-^;Vil-creERT2* mice would have tumors in 2-3 months after tamoxifen injection. All animal studies were performed in accordance with the relevant guidelines and under the approval of the Institutional Animal Care and Use Committee of Tsinghua University.

**Matrix coating for 2D culture.** For matrix coating, 48-well plates were incubated with 100 μL of Matrigel (BD Biosciences) or type I collagen (BD Biosciences) diluted in PBS (1:100) in incubator for 1 h. To form a single layer of Matrigel, we treated coverslips with 3-(trimethoxysilyl) propyl methacrylate (TMSPMA) and glass slides with octadecyltrichlorosilane (OTS). To a well of 48-well plates, 1 μL of Matrigel was dropped onto the OTS-treated glass to provide 10 μm thinckness, and TMSPMA-treated slide was put on Matrigel to form a thin layer. After immersed in 75% ethanol for 5 min, the coverslips were then removed for culture.

**Isolation of intestinal crypts and 2D culture.** Intestinal crypts were isolated as previously described [3]. Briefly, mouse intestine was cut longitudinally and washed three times with cold PBS. Villi were carefully scraped away and small pieces (5 mm) of intestine were incubated in 2 mM EDTA in PBS for 40 min on ice. These pieces were then vigorously suspended in cold PBS and the mixture was passed through 70 μm cell strainer (BD Biosciences). The crypt fraction was enriched through centrifugation (3 min at 300-400g). The crypts were embedded in Matrigel and seeded on 48-well plates. After polymerization, the crypt culture medium (Advanced DMEM/F12 supplemented with penicillin/streptomycin, GlutaMAX-I, N2, B27 and N-acetylcysteine (Invitrogen)) containing EGF (50 ng/mL, Invitrogen), Noggin (100 ng/mL, R&D) and R-spondin1 (500 ng/mL, R&D) (ENR) was added. For 2D culture, the crypts were re-suspended in the ENR-containing crypt culture medium plus 10 μM blebbistatin (Selleck) (BENR) or 10 μM Y-27632 (Selleck) for first 2 days and seeded on a thin layer of Matrigel. The 2D culture medium (the crypt culture medium containing blebbistatin (10 μM, Selleck), LDN-193189 (0.5 μM, Selleck, S2618), R-spondin1 (500 ng/mL, R&D) and CHIR-99021 (2.5 μM, Selleck, S1263) (BLRC) was added and refreshed every 2 days. Similarly, the intestinal polyps from *Apc^-/-^;Vil-creERT2* mice were cultured with the crypt culture medium containing EGF and Noggin (EN). Then, the APC KO organoids were re-suspended with the EN medium containing blebbistatin and then seeded on a thin layer of Matrigel. For seeding, the intestinal epithelial cells were acquired from the isolated crypts or the 3D organoids. For passaging, the intestinal epithelial cells in each well were suspended in 1ml cold PBS after removal of the medium and were pelleted by centrifugation (3 min at 300-400× *g*). The pelleted cells were embedded in fresh Matrigel and seeded on plates followed by addition of the ENR culture medium. For monolayer quantification, about 500 crypts were seeded each well and the survived organoids were counted 2 days later.

**Immunofluorescence.** 2D-cultured cells were fixed for 15 min with 4% paraformaldehyde at room temperature. After washed with PBS in the plate for 3 times, cells were permeabilized with 1% Triton X-100 for 15 min in the 4 ^o^C and blocked with PBT solution (3% BSA and 0.1% Triton X-100 in PBS) for 2 h. The samples were then incubated overnight with the primary antibody at 4 ^o^C. The following primary antibodies were used: rat anti-EpCam (eBioscience, 11-5791-82, 1:300), mouse anti-E-cadherin (BD, 610182, 1:300), mouse anti-N-cadherin (BD, 610920, 1:300), goat anti-Villin (Santa Cruz, SC-7672, 1:100), Rabbit anti-Ki67 (Abcam, ab15580, 1:200), goat anti-EphB2 (R&D, AF467, 1:300), rabbit anti-lysozyme (Dako, F0372, 1:200), rabbit anti-Muc2 (Santa Cruz, sc-15334, 1:300), goat anti-Chromogranin A (Santa Cruz, sc-1488, 1:300) and mouse anti ZO-1 (Invitrogen, 33-9100, 1:200). The fluorescein-labeled secondary antibodies (Life Technologies, 1:300) and 4’, 6-diamidino-2-phenylindole (DAPI) and Alexa Fluor 546 Phalloidin were applied for 1 h at room temperature. TdT-mediated dUTP nick end labeling (TUNEL) was performed by following the manufacturer’s instruction (In Situ Cell Death Detection Kit, Roche).

**RNA extraction and qRT-PCR.** Total RNA was extracted with RNeasy Mini Kit (Qiagen) according to the manufacturer’s instruction. cDNA was prepared using Revertra Ace (Toyobo). qRT-PCR was performed with TransStart Green qPCR SuperMix (Transgen Biotech) in triplicates on a LightCycler 480 (Roche) with Gapdh as the reference gene. Data were analyzed according to the ΔCT method.

**Imaging.** Images were obtained with an Olympus FV1200 Laser Scanning Microscope. Live cells were imaged using 10× objective lens and a 488 nm laser only. All the staining samples were plated on the glasses and imaged using 20× objective lens, and a 405 nm laser, a 488 nm laser, a 561 nm laser, and a 647 nm laser.

**Flow cytometry.** The 2D-cultured monolayer and 3D-cultured organoids were first suspended in cold PBS after medium discarding, pelleted by centrifugation (3 min at 300-400× *g*) and then incubated in TrypLE for 20 min at 37 ^o^C to obtain single-cell suspension. The dissociated cells were stained with propidium iodide (PI) and passed through 40 μm cell strainer (BD), and then single GFP-high cells were analyzed or sorted by flow cytometry (MoFlo XDP, Beckman). PI-negative cells were gated as GFP-positive and GFP-negative populations and analyzed with the Flowjo software.

**Statistics.** Data were expressed as the mean ± SD. Student’s *t*-test and Two-way ANOVA test were used to compare difference between two groups as indicated in the figure legends. **P* < 0.05, ***P* < 0.01, ****P* < 0.001. Statistical analysis was performed with the GraphPad Prism6 software. Each experiment was independently repeated at least three times.

**References**

1. Barker N, van Es JH, Kuipers J *et al.* Identification of stem cells in small intestine and colon by marker gene Lgr5. *Nature* 2007; **449**:1003-1007.

2. el Marjou F, Janssen KP, Chang BH *et al.* Tissue-specific and inducible Cre-mediated recombination in the gut epithelium. *Genesis* 2004; **39**:186-193.

3. Zhao B, Qi Z, Li Y *et al.* The non-muscle-myosin-II heavy chain Myh9 mediates colitis-induced epithelium injury by restricting Lgr5+ stem cells. *Nat Commun* 2015; **6**:7166.


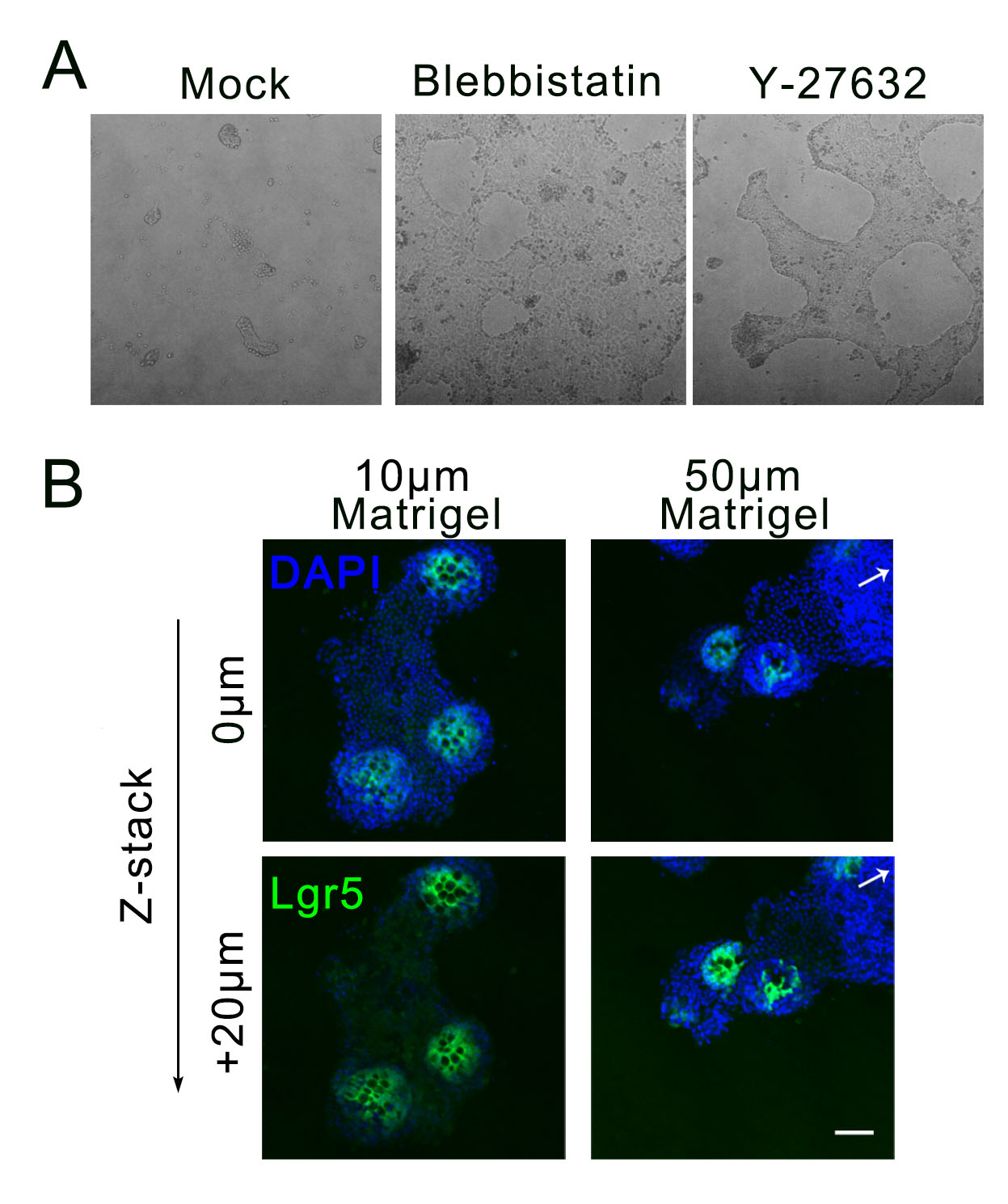


**Figure S1 Intestinal cryptic epithelium can be cultured in the Matrigel-based 2D system.** (**A**) Representative bright-field images from day 2 cultured in 10 μM blebbistatin and 10 μM Y-27632. (**B**) Z-stack confocal images of cells cultured on 10 μm or 50 μm Matrigel. Arrows mark another layer of stem cells. Scale bar, 50 μm.


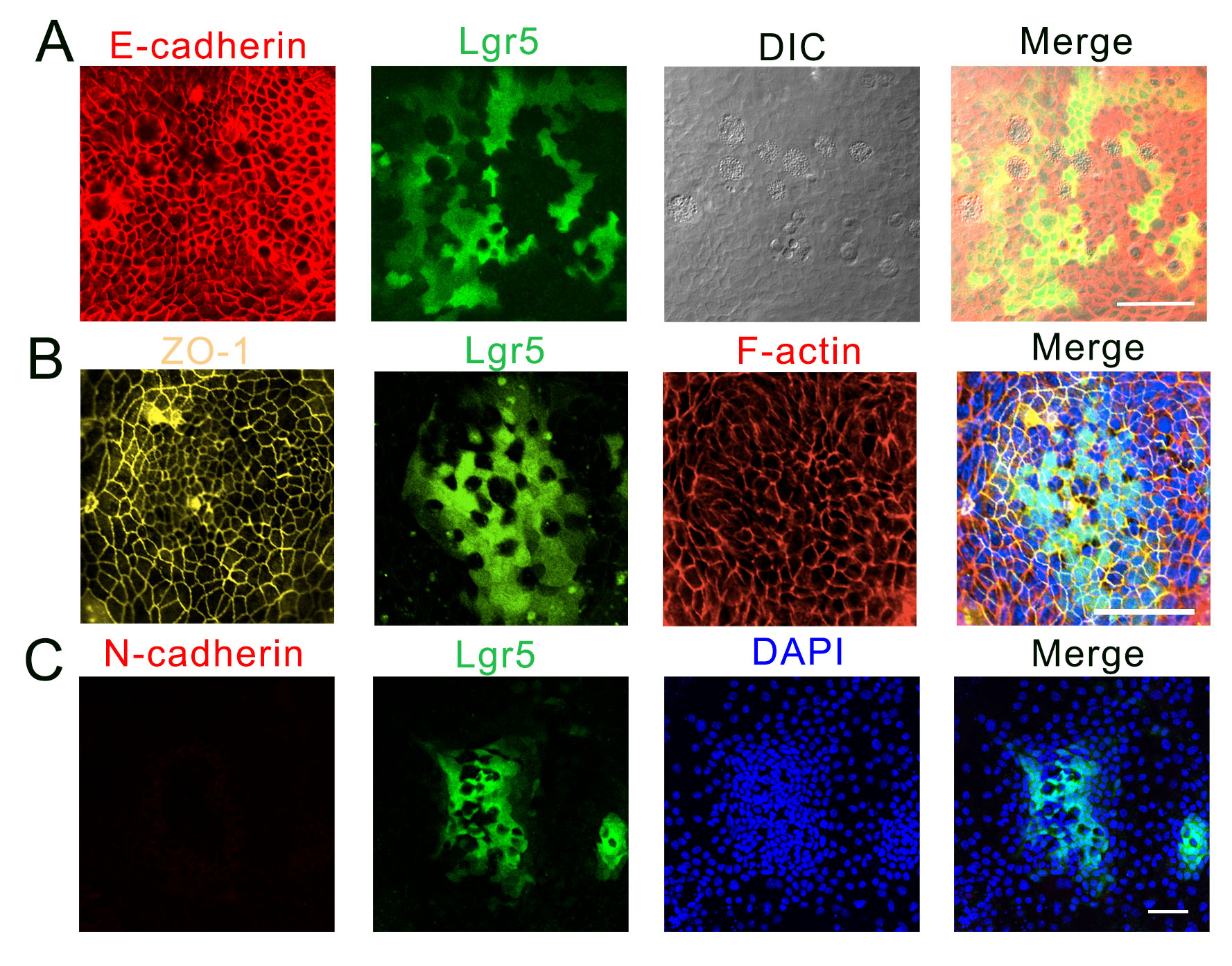


**Figure S2 The cells cultured in 2D culture system are intestinal epithelium but not mesenchymal cells.** (**A**) Confocal image of the 2D cultured cell stained for E-cadherin (red), Lgr5 (green) and DIC. (**B**) Confocal image of the 2D cultured cells stained for ZO-1 (yellow) and F-actin (red). (**C**) Confocal image of monolayers stained for N-cadherin (red), Lgr5 (green) and DAPI (blue). Scale bars, 50 μm.


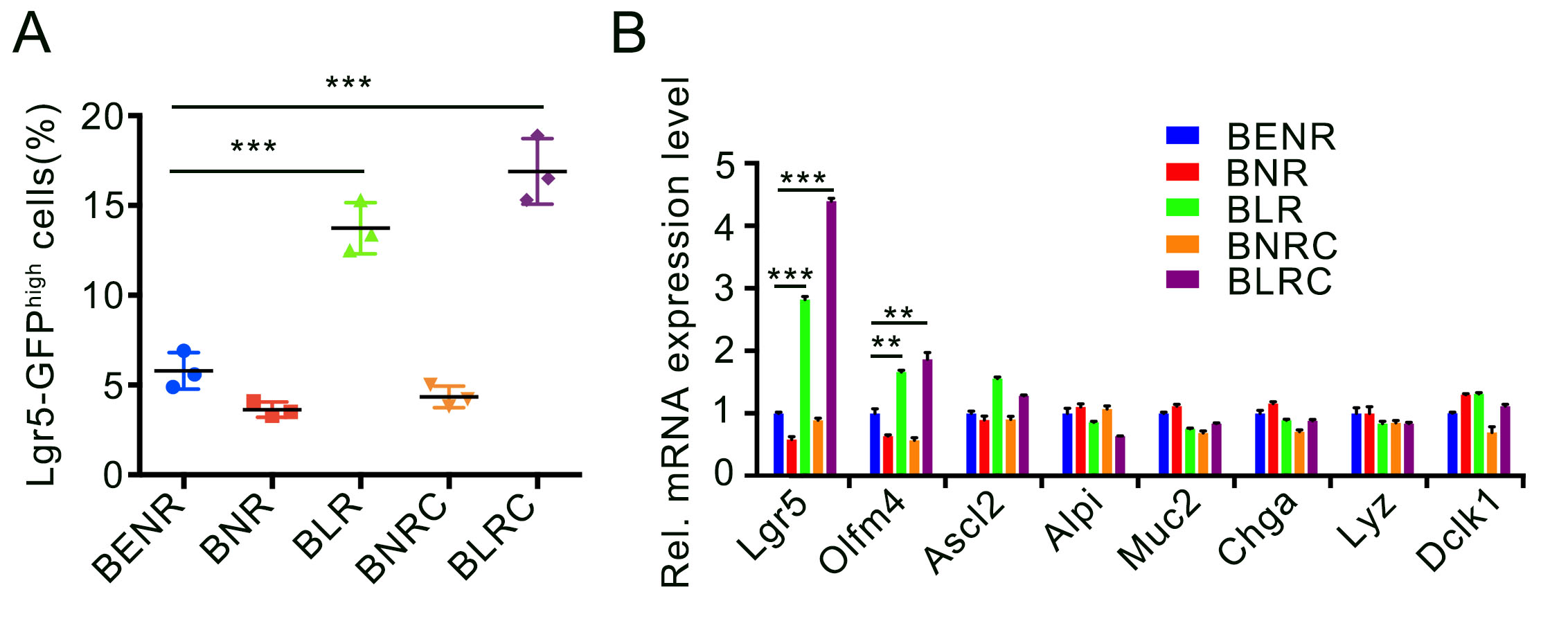


**Figure S3 Effects of different growth factors and chemicals on the monolayer culture of intestinal stem cells.**  (**A**) FACS analysis of Lgr5-GFP^high^ cells after 5 days cultured in the indicated medium. (**B**) qPCR analysis of the indicated mRNAs in the epithelial cells cultured in different medium. The data were analyzed by Two-way ANOVA test (A) or Student’s *t*-test (B) and expressed as mean ± SD. ***P* < 0.01, ****P* < 0.001.

**
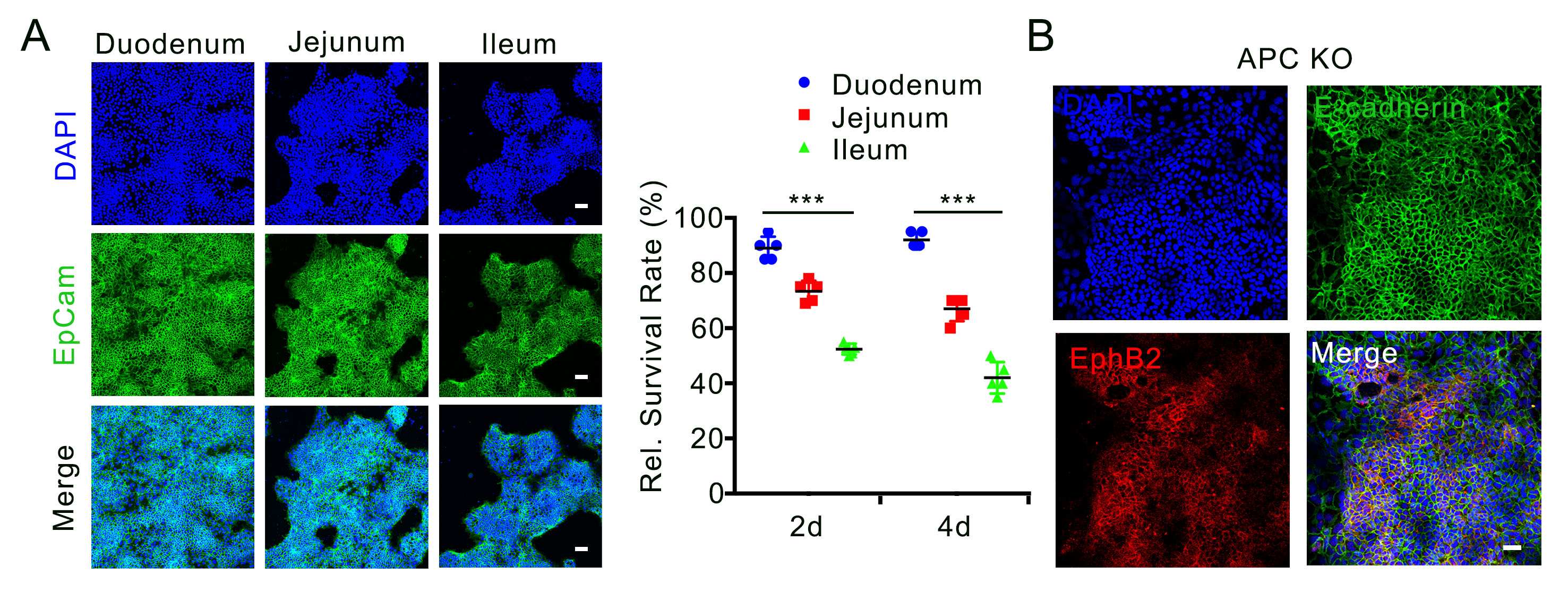
**

**Figure S4 The 2D culture system can sustain the growth of epithelial cells from different portions of small intestine and polyps obtained from APC KO mice.** (**A**) Representative confocal images show the epithelial cells from different portions of small intestinal crypts after 2 day culture. Quantification of relative survival rates is on right, and the data were analyzed by Two-way ANOVA test and expressed as mean ± SD. ****P* < 0.001. (**B**) Confocal images of the epithelium marker E-cadherin and the crypt marker EphB2 were obtained in the 2D-cultured polyps from APC KO mice. Scale bars, 50 μm.

**
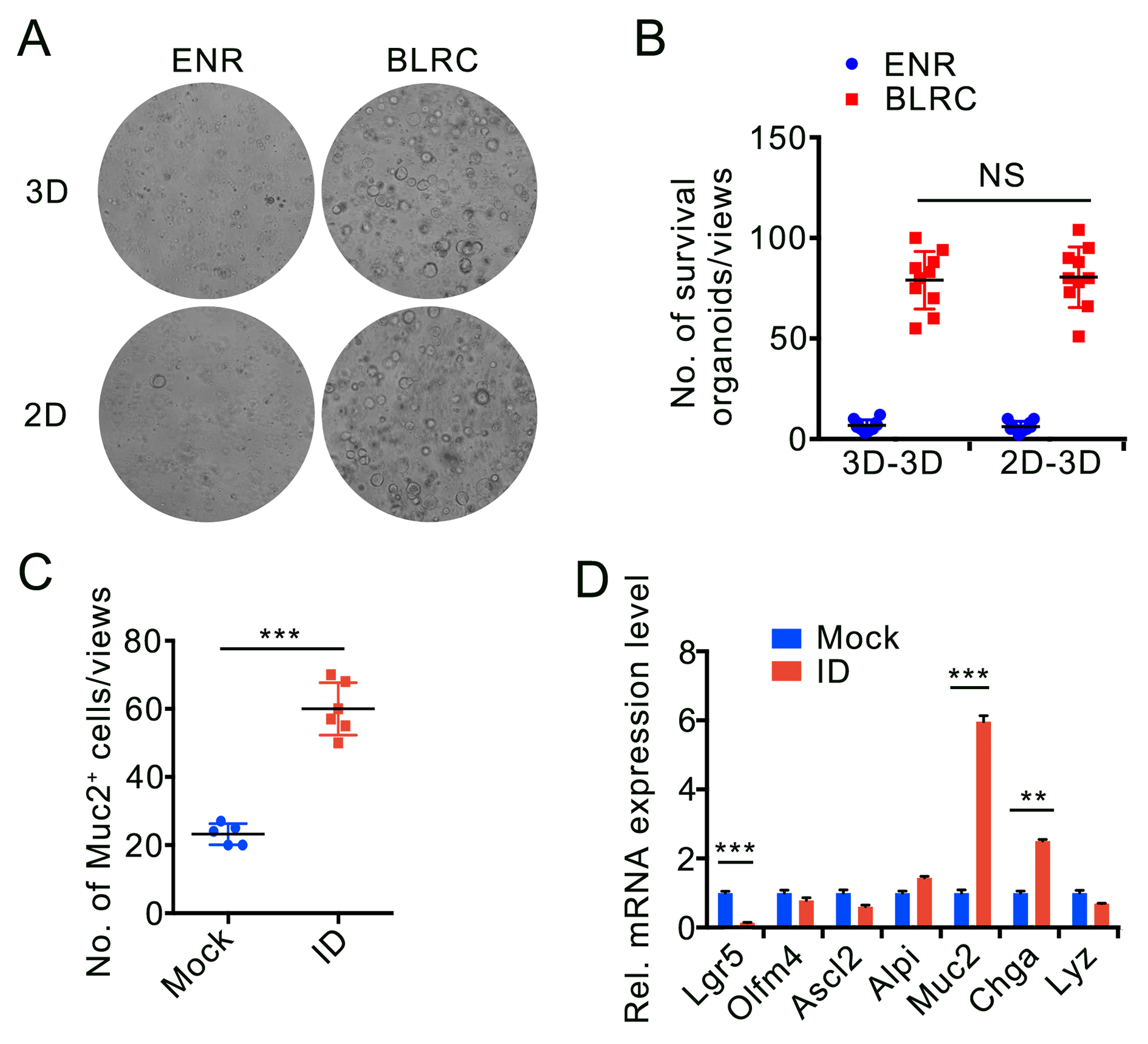
**

**Figure S5 The ISCs cultured in the 2D system retain the ability of self-renewal and differentiation.** (**A** and **B**) The epithelial cells was cultured in the 3D or 2D system for 7 days. Then, Lgr5^+^ cells were sorted and re-cultured in the 3D system with the indicated medium for 4 days before photography. Quantification of the survival organoid number is shown in **B**. (**C**) Quantification of the differentiated goblet cells (Muc2^+^) after the cells were treated with or without 2 μM IWP-2 and 10 μM DAPT (ID) for 5 days. (**D**) qPCR analysis of the mRNA expression of intestinal epithelial markers from cells pre-cultured in the 2D system (7 days) and then transferred to ENR medium with or without ID for 5 days. The data were analyzed by Two-way ANOVA test in (B) or Student’s *t*-test in (C, D) and expressed as mean ± SD. ****P* < 0.001, ***P* < 0.01 and **P* < 0.05.
