# Supplementary material for: Monolayer culture of intestinal epithelium sustains Lgr5+ intestinal stem cells
Source: Cell Discov. 2018 Jun 12;4:32. doi: 10.1038/s41421-018-0036-z (PMC5997714; doi:10.1038/s41421-018-0036-z)
Supplement: Supplementary file 2 — video legend [file 41421_2018_36_MOESM2_ESM.docx]

**Supplementary Movie** 3D reconstruction of the 2D culture of intestinal cryptic epithelium to show the monolayer cells.
